# Apoplastic interactions between plants and plant root intruders

**DOI:** 10.3389/fpls.2015.00617

**Published:** 2015-08-14

**Authors:** Kanako Mitsumasu, Yoshiya Seto, Satoko Yoshida

**Affiliations:** ^1^Graduate School of Science and Technology, Kumamoto University, Chuo-ku, Japan; ^2^Department of Biomolecular Sciences, Graduate School of Life Sciences, Tohoku University, Aoba-ku, Japan; ^3^RIKEN Center for Sustainable Resource Science, Yokohama, Japan

**Keywords:** parasitic plants, plant-parasitic nematodes, cell wall, plant cell wall degrading enzymes, strigolactone, effector

## Abstract

Numerous pathogenic or parasitic organisms attack plant roots to obtain nutrients, and the apoplast including the plant cell wall is where the plant cell meets such organisms. Root parasitic angiosperms and nematodes are two distinct types of plant root parasites but share some common features in their strategies for breaking into plant roots. *Striga* and *Orobanche* are obligate root parasitic angiosperms that cause devastating agricultural problems worldwide. Parasitic plants form an invasion organ called a haustorium, where plant cell wall degrading enzymes (PCWDEs) are highly expressed. Plant-parasitic nematodes are another type of agriculturally important plant root parasite. These nematodes breach the plant cell walls by protruding a sclerotized stylet from which PCWDEs are secreted. Responding to such parasitic invasion, host plants activate their own defense responses against parasites. Endoparasitic nematodes secrete apoplastic effectors to modulate host immune responses and to facilitate the formation of a feeding site. Apoplastic communication between hosts and parasitic plants also contributes to their interaction. Parasitic plant germination stimulants, strigolactones, are recently identified apoplastic signals that are transmitted over long distances from biosynthetic sites to functioning sites. Here, we discuss recent advances in understanding the importance of apoplastic signals and cell walls for plant–parasite interactions.

## Introduction

The apoplast, including the plant cell wall, is a compartment outside of the plasma membrane. Cell walls consist of polysaccharides, such as cellulose, hemicellulose and pectin, and structural, catalytic or signaling proteins and function as structural support for cell shapes and also as a barrier against biotic and abiotic stresses (Yokoyama and Nishitani, 2004). Plant cells meet other pathogenic organisms at the apoplast and activate the immune systems against undesirable enemies (Hückelhoven, 2007; Hématy et al., 2009; Malinovsky et al., 2014).

Plant roots typically grow in soil and face diverse microbes and pathogens that are present in the rhizosphere. Roots have a centralized vascular cylinder that contains xylem and phloem cells surrounded by endodermis, cortex and the outermost epidermal cell layers. Primary cell wall impregnation at the endodermal cell layer is called the Casparian strip that acts as a diffusion barrier in the apoplastic space. Many, but not all plants, have hypodermal/exodermal cell layers below the epidermis that are highly lignified and suberized and also act similarly to the Casparian strip (Geldner, 2013). Parasitic plants and plant-parasitic nematodes are the two major root pathogens that parasitize important crops and cause huge economic losses in agriculture globally.

Some of the parasitic plants in the family Orobanchaceae, such as *Striga* and *Orobanche*, are recognized as noxious weeds. *Striga* spp. mainly grow in sub-Saharan Africa and parts of Asia, and their preferable hosts include important agricultural crops such as sorghum, pearl millet, rice, maize, and cowpea (Spallek et al., 2013). Broomrapes, *Orobanche* and *Phelipanche* spp., are native to the Mediterranean region and Western Asia and have extended their distribution to Asia, Africa, Australia, and North and South America (Parker, 2009). For host penetration, the primary root tip of an obligate root parasitic plant is transformed into a haustorium, an invasion organ (Yoshida and Shirasu, 2012). Each haustorium attaches to the host root surface and invades the host tissues. The tip of the haustorium eventually reaches the host’s stele, and a xylem connection, called a xylem bridge, is established (Heide-Jorgensen and Kuijt, 1995). Through the xylem bridge, parasites are able to acquire water and nutrients from the host plants.

Plant-parasitic nematodes parasitize a wide range of crop plants and greatly affect agriculture with an estimated loss of almost 100 billion USD per year (Abad et al., 2008; Nicol et al., 2011). Plant-parasitic nematodes are obligate parasites and are classified according to their feeding strategy. Sedentary endoparasites are the most evolutionarily advanced and the most damaging nematode group and, therefore, the molecular mechanisms underlying successful parasitism by this group have been extensively investigated. Two of the major agricultural pest nematodes are root-knot nematodes (*Meloidogyne* spp.) and cyst nematodes (*Heterodera* and *Globadera* spp.), both of which are sedentary endoparasites. Cyst and root-knot nematodes have developed stylets, hollow mouth spears that are used to pierce the epidermis of a host root. Esophageal gland cells consisting of two subventral and one dorsal gland cell are specialized for the production and secretion of secretory proteins, whereas the stylet punctures root cell walls, injects the secretory compounds produced in the gland cells and sucks host nutrients (Hussey, 1989; Mitchum et al., 2013). After invasion into the root, nematodes migrate to establish a feeding site.

**FIGURE 1:**
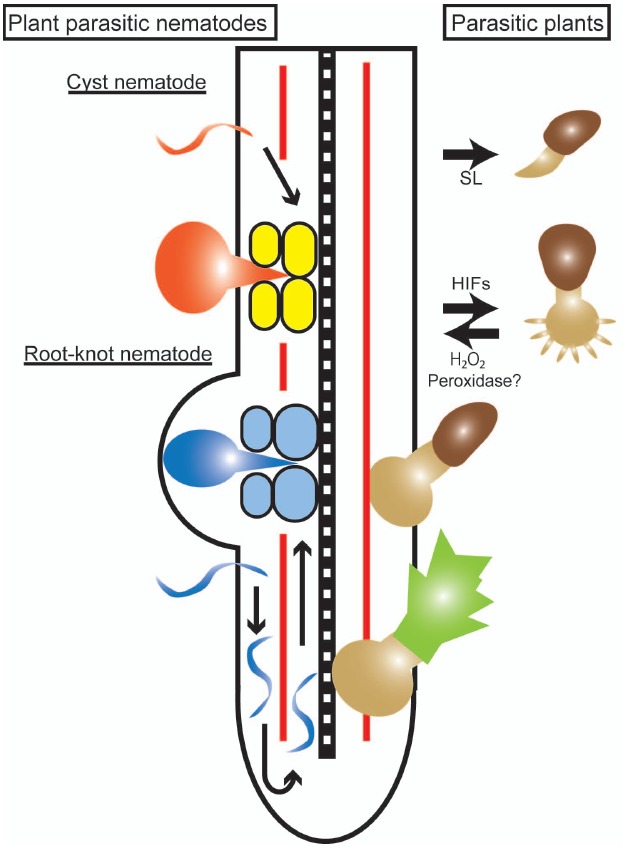
**Infection processes of parasitic plants and plant-parasitic nematodes. An illustration of a host root with the right side showing an infection by a parasitic plant (***Striga***), and the left side showing infections by cyst and root-knot nematodes.** The parasitic plant germinates in response to strigolactone (SL) and forms a haustorium in response to haustorium-inducing factors (HIFs) represented by 2,6-dimethoxy-*p*-benzoquinone. The parasite haustorium grows toward the host stele and, after reaching the host vasculature, a parasitic plant shoot begins to develop. A cyst nematode (orange) infects a host root, migrates through the cortical cells and creates a multinucleated syncytium (yellow) formed by cell fusion. A root-knot nematode (blue) infects a host root and migrates toward the root tip to avoid the Casparian strip (red line), turns and moves acropetally along the host vasculature. Giant cells (light blue) are formed by karyokinesis to provide nutrients to the nematodes.

Although these two categories of organisms are taxonomically very different, there are common features in their strategies for infecting plant cells (Figure 1). Both types of organisms are able to penetrate plant root tissues in an intra- or inter-cellular manner and grow or move toward plant vasculatures where the parasites can access the host nutrients.

In this review, we focus on the events occurring at the cell walls and the apoplastic spaces of host plant roots for these two different types of parasitic organisms.

## Cell Wall Modifications During Parasite Invasion

Plant cells form two types of cell walls, i.e., primary and secondary cell walls. In general, primary cell walls are synthesized in growing cells and are composed predominantly of cellulose, pectin, and hemicelluloses such as xyloglucans. In grass species, however, the hemicellulosic materials of primary cell walls are arabinoxylans and mixed-linkage glucans. Secondary cell walls are formed in mature cells, are laid down on the inside of the primary wall (Cosgrove, 2005) and are typically composed of cellulose, xylans and lignin, providing cellular rigidity and strength (King et al., 2011). In addition, the middle lamella, a pectin layer, fills the space between the adjacent cells and firmly adheres them (Jarvis et al., 2003). During the establishment of a parasitic infection, the cell wall barrier is broken, and apoplastic signals may function to communicate between the host and parasites (Hamann, 2014; Hofte, 2014). For breaking into plant tissues to acquire nutrients, plant cell wall degrading enzymes (PCWDEs) play important roles in pathogenic fungi, oomycetes, and bacteria (Toth and Birch, 2005; Kämper et al., 2006). Pectin degrading enzymes, including pectin methylesterase (PME), pectin lyases, and polygalacturonases, were identified in cultures of the necrotic pathogen *Botrytis cinerea* (Shah et al., 2009), and pectate lyases (PL), endoglucanases, pectin esterases, and polygalacturonases were identified in the *Phytophthora plurivora* secretome (Severino et al., 2014). Degradation of pectin layers increases the accessibility of other enzymes such as cellulases and xylanases to break down the hemicellulosic chains (Hématy et al., 2009). Large-scale activity profiling of plant pathogenic and non-pathogenic fungi revealed that the cell wall hydrolytic enzymes secreted from pathogenic fungi reflect the monocot or dicot host preferences of the tested pathogens (King et al., 2011). This result implies that fine-tuned secretion of PCWDEs contributes to fungal adaptation to the host. Against that, arrays of inhibitor proteins for fungal PCWDEs were identified from various plants and some of them are indeed involved in immune responses (Juge, 2006).

## Cell Wall Modifications by Parasitic Plants

Similar to microbe pathogens, parasitic plants are likely using PCWDEs during host root invasion. Parasitic plants form a haustorium, a unique multicellular invasion organ common to all parasitic plants. In the Orobanchaceae root parasites, globular-shaped haustoria invade host roots and form direct vascular connections with host plants, which likely enables nutrient transfer (Hibberd and Jeschke, 2001; Yoshida and Shirasu, 2012). Haustoria are formed either on the lateral side of primary or lateral roots (lateral haustoria) or at the tip of primary roots (terminal haustoria; Musselman, 1980). Haustorium development includes cell expansion and cell division of the cortical, epidermal, and pericycle cell layers (Baird and Riopel, 1984; Yoder, 2001; Ishida et al., 2011).

**FIGURE 2:**
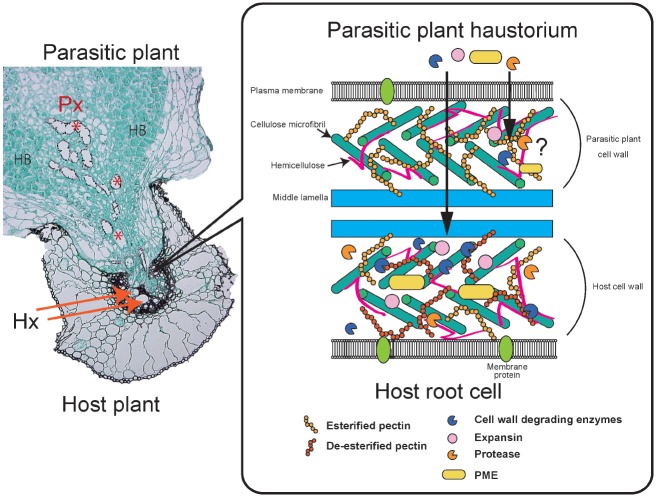
**Parasitic plant interactions with a host plant.** *Striga* infecting a rice root **(A)**. A parasitic plant haustorium penetrates a host root and connects to the vasculatures. Parasite xylem cells (Px) are indicated by red asterisks. Host xylem cells (Hx) are indicated by orange arrows. Hyaline bodies (HB) are rich in AGPs. A schematic model of parasite and host cell walls and plant cell wall degrading enzymes (PCWDEs; **B**). Parasites secrete cell wall degrading enzymes, expansins and proteases. Pectin methylesterase (PME) de-esterifies the host cell wall pectin, thereby making the pectin more accessible to polygalacturonases and pectate lyase. Host cell wall pectin is often de-esterified. PCWDEs also include xylanases targeting cell wall xylan and cellulases targeting cellulose. It is still uncertain whether parasitic plant PCWDEs affect their own cell walls.

Electron microscopic analysis revealed that intrusive cells in the haustoria of *Orobanche* and *Striga* push their way through the host epidermis and cortex to reach the host stele (Neumann et al., 1999). Biochemical and histological studies have confirmed the activities of PCWDEs and compositional changes in the host and parasite cell walls (Figure 2). For instance, cellulase, polygalacturonase and, to a lesser extent, xylanase activities were detected from *Phelipanche aegyptiaca* shoots, roots and tubers (Singh and Singh, 1993), and pectinolytic activities, such as PME and polygalacturonase, as well as peroxidase were detected in *Orobanche* seedlings (Veronesi et al., 2005, 2007; Gonzalez-Verdejo et al., 2006). Several recent transcriptome analyses confirmed expression of PCWDEs in haustoria. Highly expressed genes in *Triphysaria versicolor* haustoria analyzed by laser microdissection included homologs of pectinesterase and polygalacturonase (Honaas et al., 2013). Comparative transcriptomics of three Orobanchaceae species identified a core set of genes expressed in haustoria, including pectate lyase and cellulase (Yang et al., 2015). Immunogold labeling of PME showed that this enzyme accumulates at the cell wall and Golgi apparatus of *Orobanche cumana* intrusive cells (Losner-Goshen et al., 1998). PME may have a crucial role in parasitism since application of catechin, a PME inhibitor, results in reduced attachment of the facultative root parasite *Castilleja indivisa* to its host and has a similar effect on the stem parasite *Cuscuta pentagona* on *Arabidopsis* (Lewis et al., 2010, 2008). Pectin de-esterification by PME is a prerequisite for pectin degradation by polygalacturonase and pectate lyase. Therefore, PME may modify the host cell wall pectin to become more accessible to other pectinolytic enzymes. Indeed, a correlation between pectinolytic enzyme activity and parasite virulence was reported in *O. cumana*, highlighting the importance of enzymatic activity in parasite virulence (Veronesi et al., 2005).

Changes in cell wall compositions are also observed during the penetration process. Immunofluorescence labeling revealed that the level of pectin esterification differs between *Orobanche* spp. cell walls and host cell walls during the interaction of these two organisms (Losner-Goshen et al., 1998; Figure 2). High- and low-esterified pectins were found in the parasites, whereas only low-esterified pectins were detected in host tissues close to the interface (Losner-Goshen et al., 1998). Similarly, during stem parasite dodder penetration, antibody-based comprehensive profiles of cell wall epitopes and PCWDEs revealed the presence of highly de-esterified homogalacturonans in susceptible host cell walls coincident with high pectinolytic activity in *Cuscuta* haustoria (Johnsen et al., 2015). In the interaction between *Striga hermonthica* and sorghum, often collapsed and necrotic host cells appear at the lateral site of the invading haustorium and cytoplasmic degradation products are detected, whereas none of these changes occur at the tip of the penetrating haustorium (Neumann et al., 1999). These observations indicate that the tips of penetrating haustoria are an active site for invasion, where parasites make their way while maintaining adhesive interaction with the host cells, undergoing continuous cell division and expansion of the haustorial internal cells, and creating physical pressure on the side of the haustorium to compress and eventually collapse the host root cells. In cowpeas infected with *Striga gesnerioides*, host cells surrounding the *Striga* intrusive cells were compressed and the middle lamella was degraded (Reiss and Bailey, 1998). Although not frequent, outgrowths of haustorial cells into host root cells are observed, suggesting that the host cell walls in the interface between host and parasite are weakened (Reiss and Bailey, 1998).

High levels of expression of expansins, cell wall-loosening proteins with no known enzymatic activity, were confirmed in the haustoria of a facultative parasite, *T. versicolor*, by tissue-specific expression analysis (Honaas et al., 2013). Interestingly, β-expansin that specifically loosens monocot cell walls was expressed higher in haustoria infecting *Zea mays* roots than in infections of *Medicago truncatula*, but the expression levels of α-expansin that targets both dicot and monocot cell walls were similar in both interactions (Li et al., 2003; Honaas et al., 2013). Therefore, the generalist parasites may use distinct sets of genes for different host interactions; this hypothesis supports the model that expansins may directly act on host cell walls.

Proteases likely play roles in host plant penetration by parasitic plants. Expression of cysteine protease-encoding genes was confirmed in haustoria of *Phelipanche aegyptiaca* as well as in the stem parasite *Cuscuta reflexa* (Rehker et al., 2012). Expression of inhibitor peptides in their hosts reduces parasite infection, suggesting that the proteases function in host tissues (Rehker et al., 2012). Apart from root parasites, the transcriptome of the stem parasite *Cuscuta* revealed that abundant transcripts in the prehaustorial stage are enriched with mRNAs encoding proteins with hydrolase activity or are associated with plant cell wall structure or function (Ranjan et al., 2014).

Cell wall modifying enzymes can be secreted to degrade host cell walls; alternatively, these enzymes may also affect parasite cell walls. Hyaline bodies occupy the central region of haustoria, an observation characteristic of cells with dense cytoplasm and extracellular deposits (Visser et al., 1984; Figure 2). Development of hyaline bodies is well correlated with host compatibility, but the physiological roles of these cells still remain obscure. The cell wall of hyaline bodies has a characteristic composition enriched with arabinogalactan proteins (AGPs) and reduced de-esterified pectins (Pielach et al., 2014). Furthermore, intercellular deposits and globular ergastic bodies composed of pectins, xyloglucans, extensins and AGPs were found in the facultative parasite *Rhinanthus* (Pielach et al., 2014). Therefore, the cell wall modifying and degrading enzymes may also act to develop hyaline bodies inside the haustorium.

## Host Cell Wall Modification by Plant-Parasitic Nematodes

Plant-parasitic nematodes invade host root tissues to gain access to vasculatures. Patterns of migration and feeding site formation of cyst nematodes and root-knot nematodes are slightly different (Lambert and Bekal, 2002). Root-knot nematodes migrate intercellularly along the cortex toward the root tip and then turn around at the elongation zone to avoid the Casparian strip where the cell walls are highly lignified. The root-knot nematodes migrate up in the vascular cylinder until they reach the differentiation zone and colonialize this region (Figure 1). In contrast, cyst nematodes enter a root, migrate through cortical cells to the vascular cylinder and fuse multiple cells to form a multinucleated syncytium that functions as a feeding site (Figure 1). Root-knot nematodes modulate host cells to form multinucleated giant cells to serve as feeding sites by inducing karyokinesis without cell division. The function of the host root is significantly impaired by the intra- or inter-cellular migration of nematodes and the formation of a syncytium or giant cells that results in the destruction of root tissue structures (Rasmann et al., 2012).

**FIGURE 3:**
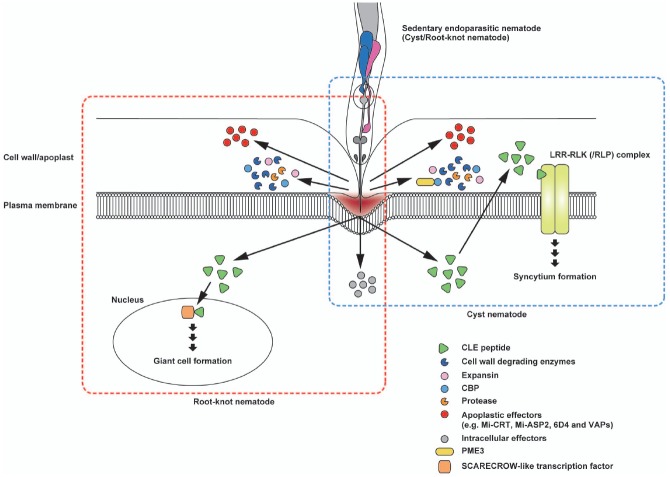
**Schematic representation of the potential interactions of secreted effector proteins with nematode feeding cells.** Sedentary endoparasites pierce the plant cell wall with their sclerotized stylets and release effector proteins from the stylet tip. Cell wall degrading enzymes, expansin and cellulose-binding protein (CBP) are beneficial for loosening the cell wall structure to facilitate nematode migration after invasion. Cyst nematode CBPs interact with plant pectin methylesterase 3 (PME3). In cyst nematodes, CLE-like peptides appear to mimic plant CLE signaling that probably promotes syncytium formation. The CLE-like peptide 16D10 from root-knot nematodes binds to two SCARECROW-like transcription factors in the host plant. Mi-CRT is involved in the inhibition of defense responses. In addition, Mi-ASP2, 6D4, and VAPs in root-knot nematodes and cyst nematodes function as apoplastic effector proteins. Illustrations framed with boxes of blue- and red-dashed lines show effector proteins and their topology for cyst nematodes and root-knot nematodes, respectively.

Plant-parasitic nematodes secrete various PCWDEs when they penetrate and migrate inside host roots (Figure 3). Genes encoding β-1,4-endoglucanase, an enzyme that degrades cellulose, were identified from two species of cyst nematodes, marking the first confirmation of PCWDEs originating from animals (Smant et al., 1998). These endoglucanases were classified as glycosyl hydrolase (GH) family five cellulases. GH5 endoglucanases were found not only in the endoparasitic genera of *Globodera*, *Heterodera*, and *Meloidogyne* (Smant et al., 1998; Béra-Maillet et al., 2000; Goellner et al., 2000; Gao et al., 2002; Abad et al., 2008) but also in many other plant-parasitic nematode genera (Danchin et al., 2010; Haegeman et al., 2011b, 2012). In the cyst nematode *Heterodera*, endoglucanases are expressed in the subventral glands in second-stage juveniles (J2) before penetration and during migration, indicating that these cellulases play an early role in the parasitism process (Smant et al., 1998; Davis et al., 2004).

In addition to cellulase activity, hemicellulolytic and pectolytic enzyme activities are detected in the secretions of plant-parasitic nematodes. *Heterodera* GH5 endoglucanases degrade hemicellulose (Gao et al., 2004). A root-knot nematode, *Meloidogyne incognita*, produces xylanases from the GH5 and GH30 families (Mitreva-Dautova et al., 2006; Abad et al., 2008; Danchin et al., 2010). Polygalacturonases from family GH28 and PL from family PL3, two types of pectolytic enzymes, were identified in *M. incognita* (Abad et al., 2008; Danchin et al., 2010; Haegeman et al., 2011a). Genes encoding pectate lyase from family PL3 were also identified in *Heterodera* and *Globodera* cyst nematodes (Popeijus et al., 2000; De Boer et al., 2002; Gao et al., 2003; Vanholme et al., 2007; Danchin et al., 2010), and these genes are expressed in the subventral esophageal glands of pre-parasitic and parasitic J2-stage nematodes. Putative arabinanases from family GH43 were also identified in *Globodera*, *Heterodera*, and *Meloidogyne* (Danchin et al., 2010). Arabinan, the substrate of arabinanase, is a main component of pectin side chains; thus, hydrolysis of arabinan may result in easier access to pectin backbones for polygalacturonases and PL (Danchin et al., 2010).

Besides PCWDEs, nematodes secrete cell wall modifying proteins. Gr-EXP, an expansin isolated from *Globodera rostochiensis*, exerts cell wall loosening activity similar to the plant expansins (Qin et al., 2004; Kudla et al., 2005). In *M. incognita*, genes encoding expansin-like proteins were identified (Abad et al., 2008; Danchin et al., 2010). Expansins may promote the accessibility of cell wall degrading enzymes to their substrates by disrupting non-covalent bonds between polysaccharide chains (Haegeman et al., 2012). Interestingly, homologous genes encoding PCWDE and expansins have not been found in free-living nematodes. Amino acid sequences of these proteins are similar to those of bacteria or fungi, indicating that these nematode genes could have been acquired from bacteria or fungi by horizontal gene transfer (Danchin et al., 2010; Haegeman et al., 2011a, 2012). Additionally, nematodes secrete a cellulose-binding protein (CBP) that contains a cellulose recognition domain and binds to cellulose but has no hydrolytic activity. CBP from the sugar beet cyst nematode *Heterodera schachtii* (Hs CBP) interacts with *Arabidopsis* pectin methyl esterase 3 (PME3) to facilitate cyst nematode parasitism (Hewezi et al., 2008). *Hs CBP* expression peaks at the parasitic J3 stage, suggesting a role during the early phases of syncytium formation (Hewezi et al., 2008). PMEs catalyze the demethylesterification of pectin in the cell wall and modify the stiffness of the cell wall (Sasidharan et al., 2011). Overexpression of *Hs CBP* slightly, but statistically significantly, increased the PME activity of *Arabidopsis*, indicating that a reduction in the methylesterification level in the cell wall pectin through Hs CBP-mediated PME3 activity induces a cell wall modification required for syncytium development (Hewezi et al., 2008). Plant-parasitic nematodes also secrete proteases. Proteases could be used to loosen the cell wall structure, thereby facilitating the migration of nematodes. In addition, proteases may degrade plant defense proteins or digest host proteins in the giant cells (Haegeman et al., 2012).

## Plant Immunity and Cell Wall Integrity

Cell walls are the physical barriers against pathogen attacks. Cell wall reinforcement is one of the primary responses against pathogen infection. The deposition of callose, a 1,3-β-glucan, is commonly observed in plant leaves and roots upon pathogen challenge (Millet et al., 2010; Voigt, 2014). In addition, alteration of cell wall integrity sensed by the host plant also is a signal to activate innate defense responses (Hématy et al., 2009). Various mutants with altered cell wall composition have a pathogen-resistant phenotype (Nühse, 2012; Malinovsky et al., 2014). For example, the *Arabidopsis* cellulose synthase mutant *cesa3* is more resistant toward powdery mildew, and defects in secondary wall-forming CESAs provide resistance against necrotrophic pathogens (Malinovsky et al., 2014). The *Arabidopsis powdery-mildew-resistant* (*pmr*) mutants also link cell wall composition and plant immunity (Nühse, 2012). The *pmr5* and *pmr6* have increased levels of unesterified pectin and enhanced powdery-mildew resistance independent of the salicylic acid (SA), jasmonic acid (JA), and ethylene (ET) pathways (Vogel et al., 2004). The disruption and overexpression of *PMR4*, a callose synthase, resulted in a resistant phenotype against powdery mildew likely with a different mechanism, indicating the involvement of *PMR4* in establishing a physical barrier and in defense signaling (Nishimura et al., 2003; Ellinger et al., 2013). In the interaction of plants and microbial pathogens, the presence of pathogens is recognized by pathogen (or microbe) associated molecular patterns (PAMPs/MAMPs), which are widely conserved molecules among microbes, through cell surface localized pattern recognition receptors. Cell wall fragments, such as oligogalacturonides (OGs), also act as damage-associated molecular patterns (DAMPs) that induce basal plant defenses (Boller and Felix, 2009; Ferrari et al., 2013). OGs are oligomers of α-1,4-linked galacturonosyl residues released from homogalacturonan, a major cell wall pectin component. Therefore, recognition of OGs through membrane-localized receptor *WALL-ASSOCIATED-KINASES* (*WAKs*) is considered a system for monitoring pectin integrity that induces a set of basal defense responses, including accumulation of reactive oxygen species and pathogenesis-related proteins (Brutus et al., 2010; Ferrari et al., 2013).

In the theory of the co-evolution of plant defense systems and pathogen infection strategies, pathogens evolved to overcome basal plant immunity by generating effectors (Jones and Dangl, 2006). Effectors are typically small proteins or peptides delivered into plant cells or the apoplast (Win et al., 2012; Stotz et al., 2014). Plants recognize the effectors directly or indirectly through resistance (R) proteins, typically nuclear-binding site (NBS) and leucine-rich repeat (LRR) motif-containing proteins, and induce effector-triggered immunity (ETI) that often results in race-specific resistance. The sequential evolution of the innate immunity systems and breakdown processes is referred to as the “zigzag” model (Jones and Dangl, 2006).

Although the above theory was based on work conducted with leaf-infecting pathogens, accumulating evidence suggests that root immune systems are, at least partially, similar to those in leaves. *Arabidopsis* roots respond to MAMP signals by promoter activation of defense-related genes and epidermal callose deposition (Millet et al., 2010). As is known in leaves, coronation, a phytotoxin and JA-Ile mimic produced by *Pseudomonas syringae* pathovars, suppresses the defense responses. This suppression relies on JA-signaling genes such as *COI1* and *MYC2*, but not on JA-SA antagonism (Millet et al., 2010). The importance of root defense is indirectly supported by the observation that MAMP responses are suppressed upon colonization by beneficial microbes (Jacobs et al., 2011; Nakagawa et al., 2011; Brotman et al., 2013). However, transcriptome analysis of roots infected with the wilt fungus *Fusarium oxysporum* revealed that gene expression patterns are distinct from those in leaves infected by the same pathogen, indicating the presence of a root-specific defense system (Chen et al., 2014). Interestingly, six quantitative trait loci (QTL) responsible for *RESISTANCE TO FUSARIUM OXYSPORUM* (*RFO*) were identified, and *RFO1* encodes a dominant allele of the *WALL-ASSOCIATED KINASE-LIKE KINASE 22* (*WAKL22*) gene, a receptor kinase similar to *WAK* (Diener and Ausubel, 2005). Collectively, these results suggest that a membrane-bound receptor kinase is involved in root defense systems.

## Host Immune Responses Against Parasitic Plants

Cell wall reinforcement is likely involved in the resistance against parasite intrusion. In some incompatible or non-host interactions with parasitic plants, “mechanical barrier” formation is reported, in which deposition of phenolic compounds at the host-parasitic plant interface was observed (Maiti et al., 1984; Goldwasser et al., 1999; Haussmann et al., 2004; Yoshida and Shirasu, 2009). Deposition of callose is recognized in resistant hosts although the physiological significance of callose deposition remains to be investigated (Lozano-Baena et al., 2007; Yoder and Scholes, 2010). In the resistant pea and *Orobanche crenata* interaction, parasite intrusion stopped at the host cortex cell layers before reaching the central cylinder and accompanied the accumulation of peroxidase, H_2_O_2_ and callose (Pérez-de-Luque et al., 2006). Protein cross-linking in the host cell walls may also play a role to block parasite invasion.

Although no molecular patterns associated with parasitic plants have been identified yet, the innate immune system is provoked by parasitic plant intrusion. Whole genome microarray of resistant (Nipponbare) and susceptible (IAC165) rice cultivars revealed that resistant interactions against *S. hermonthica* are characterized by upregulation of pathogenesis-related (PR) genes including many SA-responsive genes (Swarbrick et al., 2008). Proteome analyses identified defense-related proteins that are induced in resistant cultivars of pea and *M. truncatula* against *O. crenata* (Castillejo et al., 2004, 2009). Similarly, defense-related genes are enriched among the late-responsive genes in resistant interactions of cowpea and *S. gesnerioides*, whereas defense genes are suppressed in a compatible interaction (Huang et al., 2012a). These findings indicate the possible suppression of defense by parasitic plants in compatible interactions.

The defense-related plant hormones JA and SA likely contribute to parasitic plant resistance. Exogenous application of SA on red clover reduces *O. minor* infection with lignification of cell layers in the host endodermis (Kusumoto et al., 2007). Foliar application of benzo-1,2,3-thiadiazole-7-carbothioic acid S-methyl ester (BTH), a SA analog, or methyl jasmonate (MeJA) on rice induced increased resistance against *S. hermonthica* (Mutuku et al., 2015), indicating that SA and JA induce a systemic defense against parasitic plants. Indeed, endogenous SA and JA accumulate in rice after *S. hermonthica* infection. Transgenic rice containing a silencing construct for *WRKY45*, a key transcriptional factor in the SA signaling pathway, showed increased susceptibility against *S. hermonthica*. However, SA-deficient transgenic rice that was transformed with *NahG*, a gene encoding salicylate hydroxylase, did not show increased susceptibility, suggesting that endogenous SA is not required for *S. hermonthica* resistance. *WRKY45* regulates the JA biosynthesis genes, and application of MeJA complements *S. hermonthica* susceptibility in transgenic rice. These findings suggest synergistic effects of SA and JA on *S. hermonthica* resistance in rice (Mutuku et al., 2015).

Race-specific incompatibilities in parasitic plants and their hosts have been reported in several species (Huang et al., 2012b). Cowpea cultivar B301 contains the RSG3-301 gene for resistance to *S. gesnerioides* race SG3 but is susceptible to race SG4z (Li and Timko, 2009). The RSG3-301 gene encodes a typical R protein, consisting of a coiled-coil, NBS and LRR motifs. Knock-down of this gene results in a susceptible interaction with *S. gesnerioides* race SG3 (Li and Timko, 2009), indicating that this R protein has a crucial role in this race-specific resistance. Therefore, it is possible that a virulent *S. gesnerioides* strain may possess effectors that suppress plant immunity.

## Host Defenses Against Plant-Parasitic Nematode Infection

Endoparasitic nematodes migrate inside root tissues. Thus, physical and physiological changes within the root concomitant with nematode invasion and migration provoke defense responses in the host plant. Callose is deposited within plasmodesmata in young syncytia, and reduced callose degradation in an *Arabidopsis* β-1,3-glucanase insertion line results in smaller sizes of syncytia. Both of these observations indicate that callose deposition restricts syncytial growth in cyst nematodes (Hofmann et al., 2010).

Microarray analyses using tomato and root-knot nematodes revealed that JA- and SA-responsive genes are upregulated to different extents during nematode infection either in compatible or incompatible interactions (Bhattarai et al., 2008). Application of the SA analog BTH resulted in a slight increase in the expression of defense-related genes and resistance against nematodes (Nahar et al., 2011, 2012). SA-deficient tomato and rice overexpressing *NahG* had higher levels of nematode infection, suggesting that SA is involved in nematode resistance (Nahar et al., 2011, 2012). However, the effects of SA on nematode resistance are still controversial because others have reported that the *NahG* transgene had only partial or no effects on the NBR-LRR resistance gene *Mi-1* in mediating resistance as well as for basal resistance in tomato (Branch et al., 2004; Bhattarai et al., 2008). Foliar application of MeJA induces systemic defense responses in rice, and the rice JA biosynthesis mutants were more susceptible to *Mycosphaerella graminicola* (Nahar et al., 2011). Similarly, application of MeJA and defects in JA biosynthesis in tomato result in enhanced resistance and susceptibility to plant-parasitic nematodes, respectively (Cooper et al., 2005; Fan et al., 2014). These findings indicate that JA contributes to nematode resistance. In addition, ET and ABA also have positive and negative roles in nematode resistance, respectively (Nahar et al., 2012). The crosstalk among these hormonal signals may regulate host defenses against nematode infection.

## Apoplastic Effectors Secreted From Plant-Parasitic Nematodes

**FIGURE 4:**
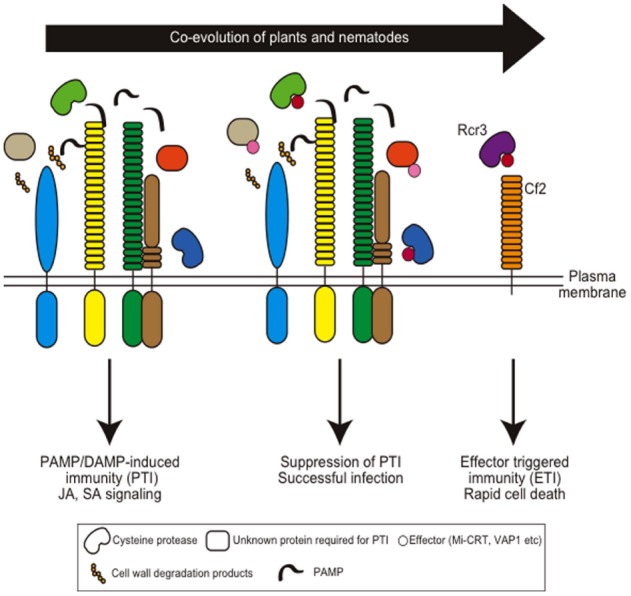
**Coevolution of host plant immunity and effectors of plant-parasitic nematodes.** Hypothetical nematode PAMPs or DAMPs are recognized by membrane-localized PAMP receptors to induce PAMP-induced immunity (PTI). Nematodes secrete apoplastic effectors that target PTI components and suppress PTI. Mi-CRT suppresses EFR-mediated defense responses when this effector is overexpressed in *Arabidopsis*. Papain-like cysteine proteases are required for nematode resistance in *Arabidopsis* and VAP effectors target these proteases. Gr-VAP1 interacts with Rcr3^pim^ in tomato and provokes Cf2-mediated defense. Note that so far no PAMP receptors have been identified that are involved in nematode resistance.

The expression of defense-related genes is suppressed during giant cell development upon root-knot nematode infection, suggesting that the host defense is shut down by nematode infection (Jammes et al., 2005; Barcala et al., 2010). Furthermore, several R-proteins have been identified for resistance against cyst- and root-knot nematodes from various plants, and this aspect is extensively reviewed elsewhere (Williamson and Kumar, 2006; Kaloshian et al., 2011). These observations suggest that the interaction between plants and plant-parasitic nematodes follows the zig-zag model (Figure 4).

Indeed, cyst and root-knot nematodes produce effector proteins and inject them into the target cells during a successful infection. Effector proteins act to modify the host–plant signaling pathway to syncytium or giant cell formation and to suppress plant defense responses that are activated by disruption of cell structure and secreted components from nematodes. In several cases, targets of effector proteins and sensors of exogenous nematode-derived substances are known to be present in the apoplast of host roots (Figure 3). Two *M. incognita* secreted proteins, Mi-ASP2, an aspartic protease-like protein, and 6D4 protein, are secreted into the apoplast along the giant cells during the migratory pre-parasitic J2 early sedentary juvenile stages, although their function during the early stage of parasitism has not been characterized (Vieira et al., 2011). Calreticulin, a Ca^2+^-binding chaperone from the root-knot nematode *M. incognita* (Mi-CRT), localizes to the apoplastic space of the giant cells (Jaubert et al., 2005). Mi-CRT is produced in the subventral and the dorsal esophageal gland cells during the migratory and sedentary stages, respectively. After secretion, Mi-CRT localizes outside of the nematode stylet tip and accumulates along the cell walls of the giant cells (Jaubert et al., 2005). Ectopic overexpression of Mi-CRT in *Arabidopsis thaliana* increases susceptibility to *M. incognita* in terms of gall formation, as well as to the pathogenic oomycete *Phytophthora parasitica*. Mi-CRT overexpression suppresses the expression of defense marker genes and callose deposition upon treatment with elf18 peptide, a well-known PAMP signal derived from acetylated, elongation factor Tu (EF-Tu), suggesting that Mi-CRT suppresses PAMP-mediated plant defense responses (Jaouannet et al., 2013). The expression of an enhanced green fluorescence protein (eGFP)-fusion construct in tobacco leaves confirmed that the overexpressed Mi-CRT localizes mainly to apoplasts, but it also localizes to the endoplasmic reticulum (ER) and Golgi network. Therefore, it remains possible that ectopic expression of Mi-CRT in the ER rather than in the apoplast suppresses elf18-induced defense responses as the elf18 receptor EFR-mediated pathway is highly sensitive to ER protein dysfunction (Tintor and Saijo, 2014).

Apoplastic venom allergen-like proteins (VAPs) are secreted proteins uniquely conserved among plant- and animal-parasitic nematodes. The expression of VAP from the cyst nematode *G. rostochiensis* (Gr-VAP1) was detected in the subventral esophageal glands at the infective juvenile stage (Lozano-Torres et al., 2012). Recent analyses revealed that suppression of Gr-VAP1 expression by dsRNA in *G. rostochiensis* results in reduced infection, and ectopic overexpression of Gr-VAP1 and VAPs from the *Arabidopsis*-infective cyst nematode *H. schachtii* (Hs-VAP1 and Hs-VAP2) enhanced infection by *H. schachtii* (Lozano-Torres et al., 2014). Moreover, *Arabidopsis* plants expressing the Hs-VAPs had enhanced susceptibility to multiple fungal and oomycete pathogens, suggesting that VAPs are effectors that modulate basal immunity in host plants. The delivery of Gr-VAP1 coincides with the enzymatic breakdown of plant cell walls by migratory nematodes (Lozano-Torres et al., 2014). Thus, these effectors may suppress host defenses activated by cell wall breakdown products during nematode infection and migration. Interestingly, in tomato, Gr-VAP1 targets the apoplastic cysteine protease Rcr3^pim^ from *Solanum pimpinellifolium* and provokes receptor-like protein Cf2-mediated defense responses by perturbing Rcr3^pim^ function (Lozano-Torres et al., 2012). This strategy is similar to the effector protein Avr2 secreted from the fungal pathogen *Cladosporium fulvum* that also induces a Cf2-mediated defense response by targeting Rcr3^pim^ (Lozano-Torres et al., 2012). This is one example of an effector that suppresses PAMP-triggered immunity and that has evolved to induce ETI in a particular host species (Figure 4).

Last year, a novel group of hyper-variable extracellular effectors (HYP) from *Globodera pallida* was discovered (Eves-van den Akker et al., 2014). Unlike general nematode effectors, the *Gp-HYP* genes are expressed in the amphid sheath cells of parasitic females rather than in the esophageal gland cells. Gp-HYP effector proteins secreted from the amphids were detected in the apoplast adjacent to the feeding site. *In planta* RNAi-mediated knock down of all members of the effector family reduced the level of successful parasitism, although the functional role and the significance of the remarkable variability of the *HYP* gene family is under discussion.

## CLAVATA3/ESR-Related (CLE)-Like Peptides as Effectors of Plant-Parasitic Nematodes

Both cyst and root-knot nematodes produce CLAVATA3/ESR-related (CLE)-like peptides as effector proteins (Figure 3). CLE peptides act like peptide hormones in plants by interacting with the apoplastic side of a complex of LRR receptor-like kinases (LRR-RLKs) and LRR-receptor-like protein (RLP) that regulate apical and cambial meristem maintenance (Betsuyaku et al., 2011). Unlike cell wall modifying enzymes, nematode CLE-like peptides are thought not to be acquired by horizontal gene transfer but to have evolved as mimics of the host peptides by convergent evolution (Haegeman et al., 2011a). CLE peptides from the soybean cyst nematode *Heterodera glycines* are transported from the cytoplasm to the host cell apoplast to function as plant CLE mimics (Wang et al., 2010). Thus, nematode CLEs mimic plant CLEs by interacting on the apoplastic side of the LRR-RLKs complex. The ectopic expression of *HgSYV46*, a gene encoding the *H. glycines* CLE peptide, in an *Arabidopsis clavata* (*clv*)*-3* mutant partially or fully rescues the mutant phenotype. Additionally, HgSYV46 transgenic plants mimic the *wuschel* (*wus*)-like phenotype and the short-root phenotype in shoot apices and roots, respectively, similar to the phenotype reported in plants overexpressing *AtCLV3* (Wang et al., 2005). Localization of HgSYV46 in syncytia was demonstrated using an affinity-purified anti-HgCLE peptide antibody (Wang et al., 2010). The number of female nematodes are significantly reduced in *Glycine max* inoculated with *HgSYV46*-knockdown *H. glycines*, and in *A. thaliana* expressing the *Hssyv46* (a *Hgsyv46* gene homolog in *Heterodera schachtii*) RNAi construct inoculated with *H. schachtii* (Bakhetia et al., 2007; Patel et al., 2008). Similar to plant intrinsic CLV3 peptide signaling, nematode CLE signaling genetically requires CLV2 and CORYNE (CRN)/SUPPRESSOR OF LLP1 2 (SOL2; Replogle et al., 2011). These results indicate that *Heterodera* CLEs are functionally similar to the CLV3 peptide and may play a role in the formation of syncytia. The *CLE*-like genes of a potato cyst nematode, *G. rostochiensis*, encode secreted proteins containing multiple CLE motifs (Lu et al., 2009). Ectopic *GrCLE* gene expression rescues the *Arabidopsis clv3-2* mutant phenotype. Furthermore, overexpression of *GrCLE* genes as well as exogenous application of synthetic GrCLE peptides result in a short-root phenotype in *Arabidopsis* roots and potato hairy roots, suggesting that GrCLE peptides also have an activity similar to plant and *Heterodera* CLE peptides. Therefore, the evolution of multiple CLE motifs may allow the generation of functional diversity in nematode CLE proteins, thereby facilitating parasitism (Lu et al., 2009). In *M. incognita*, the *16D10* gene encodes a CLE-like peptide precursor protein that is processed to a mature peptide consisting of 13 amino acids. Silencing of *16D10* expression in nematodes by dsRNA ingestion or in an *A. thaliana* host expressing dsRNA as a transgene resulted in reduced nematode infectivity (Huang et al., 2006). Overexpression of *16D10* stimulated root growth but could not rescue the *clv3-1* phenotype in *Arabidopsis*. The 16D10 was found to interact with two SCARECROW-like (SCL) transcription factors that are members of the GRAS family implicated in root development (Huang et al., 2006). The 16D10 peptide seems to regulate root cell differentiation to form feeding cells, but its detailed function has not been determined yet. CLE peptides are strong effector candidates that modulate root development. Further investigations will reveal the similarity and differences of CLE peptide signaling between cyst nematodes and root-knot nematodes.

## Apoplastic Signals for Parasites

In the interaction between host plants and microbes, host-secreted compounds act as chemical cues to establish the interaction. For example, in the symbiotic interaction between legume plants and nitrogen-fixing rhizobia, root-released flavonoids activate the production of rhizobial nod factors, lipochitin oligosaccharides, that are perceived by plants to start symbiotic programs (Hassan and Mathesius, 2012). Parasitic plants recognize host-secreted germination stimulants, strigolactones (SLs) and related compounds, that have recently attracted attention as apoplastic signaling molecules. Haustorium formation by parasitic plants also is provoked by cell-wall related compounds.

## SLs as Apoplastic Signals in Plants and the Rhizosphere

**FIGURE 5:**
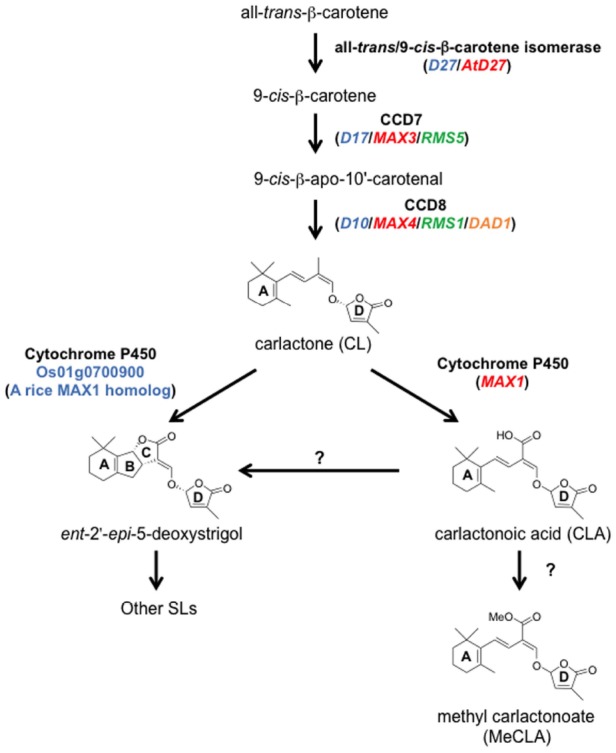
**The SL biosynthetic pathway.** CL is produced from all-*trans*-β-carotene by three enzymes, D27, CCD7, and CCD8. CL is oxidized by MAX1 into CLA, and then CLA is converted to the methyl ester derivative, MeCLA, in *Arabidopsis*. CLA is converted to strigolactones such as *ent*-2′-*epi*-5-deoxystrigol in rice. In addition, one of the MAX1 homologs in rice, Os01g0700900, can catalyze the conversion from CL to *ent*-2′-*epi*-5-deoxystrigol by a single enzymatic reaction. *Ent*-2′-*epi*-5-deoxystrigol might be converted into other SLs. Blue, red, green, and orange letters indicate genes of rice, *Arabidopsis*, pea, and petunia, respectively.

The germination stimulants, SLs, belong to a class of terpenoid lactones with the tricyclic lactone part (ABC-ring) connected to another lactone (D-ring) through an enol ether bridge (Figure 5). SLs were first reported in 1966; strigol, a natural SL, was isolated from cotton root exudates as a compound that stimulates seed germination of the obligate parasite *Striga lutea* (Cook et al., 1966). After this initial discovery of strigol, a variety of related compounds were identified from various plant species, and these compounds are collectively called SLs (Yoneyama et al., 2010). Later, SLs were rediscovered as inducers for the hyphal branching of arbuscular mycorrhizal (AM) fungi, thereby facilitating the uptake of inorganic nutrients to the host plant (Akiyama et al., 2005), and as a novel class of plant hormone that inhibits shoot branching. The *more axillary growth* (*max*) mutants in *Arabidopsis*, the *ramosus* (*rms*) mutants in pea and a few of the *dwarf* (*d*) mutants in rice have a greater degree of shoot branching and contain mutations in either the SL biosynthesis genes or the SL signaling genes (Gomez-Roldan et al., 2008; Umehara et al., 2008). More recently, SLs were demonstrated to have multiple hormonal roles in diverse developmental processes (Seto et al., 2012; Brewer et al., 2013; Ruyter-Spira et al., 2013; Waldie et al., 2014). Thus, SL functions can be classified into two groups: rhizosphere signals (allelochemical) to communicate with symbionts and parasites and endogenous hormones to regulate various plant developmental processes.

**FIGURE 6:**
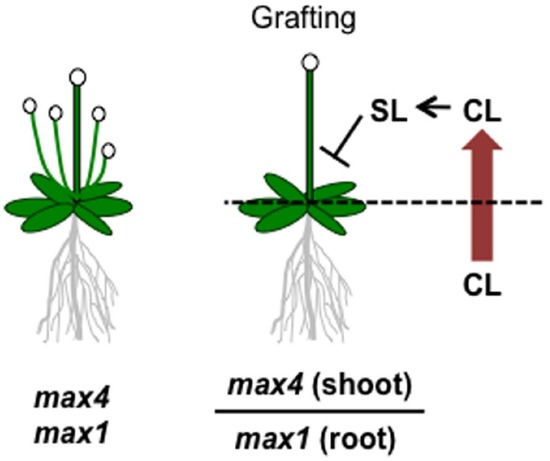
**Graphical summary of a grafting experiment using the ***Arabidopsis max4*** and ***max1*** mutants**. The shoot-branching phenotype of *max4* was restored by grafting onto *max1* roots. The model proposes that CL biosynthesized in the *max1* mutant roots is transported to the shoots, converted to SL, and inhibits shoot branching.

Although SLs are known to be carotenoid-derived compounds (Matusova et al., 2005), their biosynthetic pathway has been a long-standing question until recently. Analyses of shoot-branching mutants from several plant species identified four key enzymes in the SL biosynthetic pathway: DWARF27 (D27), a carotenoid cleavage dioxygenase7 (CCD7/MAX3), a carotenoid cleavage dioxygenase8 (CCD8/MAX4) and a cytochrome P450 (CYP711A1/MAX1; Booker et al., 2004; Arite et al., 2007; Lin et al., 2009; Crawford et al., 2010). Alder et al. (2012) demonstrated the biochemical functions of three of these enzymes, D27, CCD7, and CCD8, using *in vitro* biochemical reactions (Alder et al., 2012). As a result, carlactone (CL), a key biosynthetic intermediate with an SL-like carbon skeleton including the D-ring, was identified as a product of the sequential reaction of these three enzymes with all-*trans*-β-carotene (Figure 5). CL was further demonstrated to be an endogenous biosynthetic precursor of SL (Seto et al., 2014). In a series of grafting experiments using the *Arabidopsis max* mutants, the shoot branching phenotype of the *max4* (*ccd8*) mutant was restored by grafting onto the *max1* (*cyp711A1*) mutant rootstock (Booker et al., 2005), suggesting that MAX1 is a downstream component of CCD8. Interestingly, the *Arabidopsis max1* mutants accumulate extremely high levels of endogenous CL. Thus, we predicted that CL is the substrate of the MAX1 enzyme. Indeed, MAX1 was experimentally shown to convert CL into its C-19 oxidative product, carlactonoic acid (CLA) through three consecutive oxidation steps (Abe et al., 2014; Figure 6). Furthermore, among the five copies of the *CYP711A1* family of genes in rice, one gene product catalyzes several multi-step reactions converting CL into *ent*-2′-*epi*-5-deoxystrigol, a bioactive SL (Zhang et al., 2014; Figure 6).

As a rhizosphere signal, root-synthesized SL should be secreted into the rhizosphere through the plant apoplast. Experiments in which shoots of SL-biosynthesis mutants were grafted on wild type rootstocks and recovered the shoot branching phenotype demonstrated that this endogenous hormonal signal is transmissible long distances from roots to shoots (Booker et al., 2005; Ongaro and Leyser, 2008). This result strongly suggests that root-synthesized SL or related compounds can be transported to aerial tissues to control shoot branching. In fact, SLs have been detected in the xylem sap of *Arabidopsis* and tomato (Kohlen et al., 2011, 2012). Moreover, the results of the *max1*-shoot and *max4*-root grafting experiment described above raises another important issue; that is, the SL biosynthetic intermediate in the pathway between MAX4 and MAX1 is the transmitted form of the long-distance SL signal (Booker et al., 2005). Considering the recent discovery of CL as a product of CCD8 and a substrate of MAX1, CL is hypothesized to be the transmissible intermediate that moves from roots to shoots (Figure 5), although experimental confirmation is still necessary. On the other hand, CL is biologically inactive for shoot branching inhibition because the CL over-accumulating *max1* mutant still has the branched phenotype, and CL rescues the phenotype of the *max4* mutant but not that of the *max1* mutant (Scaffidi et al., 2013; Seto et al., 2014). These results indicate that CL is converted into an active compound after transport to the aerial parts of the plant (Figure 5). D27, CCD7, and CCD8 localize in plastids and are expressed in root tips, the root cortex, and hypocotyls (Sorefan et al., 2003; Booker et al., 2004; Auldridge et al., 2006; Arite et al., 2007; Lin et al., 2009; Waters et al., 2012a), whereas *MAX1* is expressed in vascular-associated tissues such as the cambial region and xylem-associated parenchyma in shoots and roots (Booker et al., 2005). These different expression patterns imply that MAX1 oxidizes CL during loading or unloading from the xylem (Waldie et al., 2014). In addition to CL and CLA, the methyl ester derivative of CLA (MeCLA) was also identified from *Arabidopsis* root extracts (Figure 5; Abe et al., 2014; Seto et al., 2014). Interestingly, these CL-related chemicals are able to stimulate the germination of parasitic plant seeds (Abe et al., 2014). Recently, heliolactone, a derivative of MeCLA, was identified from sunflower root exudates as a germination stimulant for seeds of root parasitic plants (Ueno et al., 2014). These results indicate that the germination of parasitic plant seeds is stimulated by chemicals secreted from host plant roots that include not only typical SLs but also CL-related compounds of more diverse structure.

The ATP-binding cassette (ABC) type transporter, PDR1, characterized as an SL-exporter in petunia, provided a clue for understanding SL transport (Kretzschmar et al., 2012). In the *pdr1* mutant, exudation of SL from roots is highly reduced compared with that from wild type roots, resulting in reduced symbiotic interactions with AM fungi and reduced germination rates of the parasitic plant *Phelipanche ramosa*. Moreover, the *pdr1* mutant has an increased branching phenotype, possibly due to a decrease in SL transported from roots to shoots. These results strongly imply that controlling SL exudation can be a powerful tool for regulating parasitic plant infections, AM fungal symbioses and plant architecture. Identification of SL transporters from various plant species and detailed substrate-specificity analyses will be required to reveal the SL secretion mechanism in plants.

Although how parasitic plants recognize SL is still unknown, a receptor candidate protein and its partner proteins for the shoot branching inhibition pathway were identified from SL-insensitive mutants. *MAX2*/*D3*/*RMS4* encodes an F-box protein, the substrate recognition subunit of the ubiquitin E3 ligase, Skp, Cullin, and F-box containing complex (SCF complex; Stirnberg et al., 2002; Ishikawa et al., 2005; Johnson et al., 2006). D14 encodes an α/β-fold hydrolase family protein (Arite et al., 2009). Through biochemical and biological analyses, D14 is proposed to be an SL receptor protein (Arite et al., 2009; Hamiaux et al., 2012; Kagiyama et al., 2013; Zhao et al., 2013). D14 was originally identified from the rice *dwarf14* mutant (Arite et al., 2009), and later its orthologous genes were identified in *Arabidopsis* (*AtD14*) and petunia (*DAD2*, Hamiaux et al., 2012; Waters et al., 2012b). D14/AtD14/DAD2 has conserved catalytic triad residues that are important for catalyzing the hydrolase reaction; in fact, these enzymes can hydrolyze a synthetic SL analog, GR24 (Hamiaux et al., 2012; Kagiyama et al., 2013; Zhao et al., 2013). Additionally, D14 can interact with D53, a recently characterized repressor protein in the SL pathway, in an SL-dependent manner (Jiang et al., 2013; Zhou et al., 2013). Furthermore, D53 protein is degraded through the 26S proteasome pathway in a manner requiring the SCF^D3^ F-box protein (Jiang et al., 2013; Zhou et al., 2013). Interestingly, MeCLA interacts directly with AtD14 protein *in vitro*, whereas CL and CLA do not (Abe et al., 2014). These results suggest that, in addition to SL, MeCLA may act as an active hormone in the shoot branch inhibition pathway.

A database search of the *S. hermonthica* ESTs showed that this species has at least one *D14* homologous gene and multiple copies of a closely related gene, *HTL/KAI2* (Toh et al., 2014). Possibly these proteins are involved in the SL perception step in parasitic plant seeds. In addition to the *D14*-related genes, *S. hermonthica* has a *D3*/*MAX2*/*RMS4* homologous gene, *ShMAX2*. The expression of *ShMAX2* in the *Arabidopsis max2* mutant complements its branching and hypocotyl elongation phenotypes (Liu et al., 2014). These results suggest that components of the SL perception system in non-parasitic plants are conserved in parasitic plants. However, functional confirmation is necessary to clarify whether parasitic plant seeds recognize SL for their germination through these SL perception systems. The presence of SL biosynthetic genes such as *CCD7* and *CCD8* in the *S. hermonthica* EST was reported, and germination assay experiments using *in vitro* cultured *Striga* extracts suggested the presence of endogenous SL in *Striga* (Liu et al., 2014). If *Striga* plants also produce SL, it will be very interesting to know how endogenous and exogenous SLs are distinguished.

## Haustorium-Inducing Signals in Parasitic Plants

Haustorium formation in Orobanchaceae parasitic plants are also medicated by apoplastic compounds, commonly called haustorium-inducing factors (HIFs), which are likely derived from cell wall degradation. The first identified HIF, 2,6-dimethoxy-*p*-benzoquinone (DMBQ), was characterized from sorghum root extracts to induce haustorium formation in a facultative parasite *Agalinis purpurea* and an obligate parasite *Striga asiatica* (Chang and Lynn, 1986). Later, several structurally related quinones, phenolic acids and flavonoids were found to have HIF activity, including *p*-coumaric acid, syringic acid and vanillic acid, major components of lignin, a cell wall polymer. Furthermore, flavonoids and quinones within a certain redox range were shown to have HIF activities in *S. asiatica* (Smith et al., 1996; Albrecht et al., 1999; Figure 1). Catalase, an enzyme that catalyzes the decomposition of H_2_O_2_, terminates the HIF activity of syringic acid but not DMBQ. Thus, a hypothesis was proposed that host-released cell wall phenolics are oxidized and converted into a signal for invasion. Indeed, *S. asiatica* seedlings accumulate H_2_O_2_, and apoplastic peroxidases identified from the parasite are able to convert syringic acid to DMBQ *in vitro*, although these peroxidases may not be specific to parasitic plants (Kim et al., 1998; Keyes et al., 2000, 2007). However, two fundamental questions are still unsolved. How are the host cell wall components released to the rhizosphere? And why do parasitic plants not respond to their own cell walls? PCWDEs secreted from parasitic plants may activate the release of cell wall phenolics from host roots and provoke haustorium development, although molecular evidence is still lacking. Finding that quinone oxidoreductase (*TvQR1*) from the facultative parasite, *T. versicolor*, is important for haustorium formation supports the hypothesis that redox regulation of quinones is a key step for haustorium induction (Bandaranayake et al., 2010). High rates of natural polymorphisms in *TvQR1* may contribute to the responses to diverse quinones that exist in various host plants (Ngo et al., 2013).

## Conclusions and Future Perspectives

Parasitic plants and plant-parasitic nematodes are both root invaders and share some commonality in their invasion systems. Notably, breaking the cell wall barrier and perceiving the preceding apoplastic signals are the most crucial steps for infection. In obligate parasitic plants in the Orobanchaceae family, the start of their life cycle is already under the control of the host-derived apoplastic/rhizosphere signal, SL and related compounds. Furthermore, parasitic plants also recognize apoplastic signals likely derived from host cell walls for haustorium formation. However, the role of cell wall degradation in producing HIFs remains to be discovered. In plant-parasitic nematodes, the rhizosphere signals required for host root recognition remain to be identified with difficulty, though chemotaxis has been suggested to be the primary means by which plant-parasitic nematodes locate host roots (Zhao et al., 2000; Reynolds et al., 2011; Dong et al., 2014). Both parasitic plants and plant-parasitic nematodes use PCWDEs to penetrate and reach host plant steles. Pectinolytic enzymes, including PME, polygalacturonase and pectate lyase are commonly used by parasitic plants and plant-parasitic nematodes to degrade primary cell walls and middle lamella, suggesting that these enzymes may contribute to the intercellular invasion by the parasites. Plant-parasitic nematodes acquired PCWDEs by horizontal gene transfer. In parasitic plants, horizontal gene transfer from host plants can occur (Yoshida et al., 2010). Cell wall degradation from parasitic invasion may act as a defense-activating signal. The identification of cyst nematode effectors that suppress the surface-localized receptor-mediated defense response implies that the effectors may have evolved to overcome DAMP-induced immunity. Several nematode effector proteins were found to mimic or inhibit the elaborate signaling networks of host cells to suppress host immune responses or facilitate reconstruction of their feeding cells, but this pathway is still far from being completely identified. Detailed analysis of nematode effector functions and their target sites will aid efforts to confer nematode resistance to host crops. Effector proteins have not been reported in parasitic plants, but the accumulating evidence for race-specific relationships between hosts and parasites suggests the existence of parasitic plant effectors to suppress host defenses. The identification of more effectors and secreted proteins, including PCWDEs, will reveal how parasites successfully invade plants and how host plants defend themselves. This approach will help to determine how such sophisticated interaction systems are established using the coevolution processes of host and parasites. Furthermore, more detailed characterization of the parasite invasion and host defense systems will contribute to the development of new control methods for these noxious agricultural pathogens.

### Conflict of Interest Statement

The authors declare that the research was conducted in the absence of any commercial or financial relationships that could be construed as a potential conflict of interest.
